# Development and Validation of a High‐Quality Composite Real‐World Mortality Endpoint

**DOI:** 10.1111/1475-6773.12872

**Published:** 2018-05-14

**Authors:** Melissa D. Curtis, Sandra D. Griffith, Melisa Tucker, Michael D. Taylor, William B. Capra, Gillis Carrigan, Ben Holzman, Aracelis Z. Torres, Paul You, Brandon Arnieri, Amy P. Abernethy

**Affiliations:** ^1^ Flatiron Health New York NY; ^2^ Genentech South San Francisco CA

**Keywords:** Mortality data, electronic health records, data quality, external validation, oncology

## Abstract

**Objective:**

To create a high‐quality electronic health record (EHR)–derived mortality dataset for retrospective and prospective real‐world evidence generation.

**Data Sources/Study Setting:**

Oncology EHR data, supplemented with external commercial and US Social Security Death Index data, benchmarked to the National Death Index (NDI).

**Study Design:**

We developed a recent, linkable, high‐quality mortality variable amalgamated from multiple data sources to supplement EHR data, benchmarked against the highest completeness U.S. mortality data, the NDI. Data quality of the mortality variable version 2.0 is reported here.

**Principal Findings:**

For advanced non‐small‐cell lung cancer, sensitivity of mortality information improved from 66 percent in EHR structured data to 91 percent in the composite dataset, with high date agreement compared to the NDI. For advanced melanoma, metastatic colorectal cancer, and metastatic breast cancer, sensitivity of the final variable was 85 to 88 percent. Kaplan–Meier survival analyses showed that improving mortality data completeness minimized overestimation of survival relative to NDI‐based estimates.

**Conclusions:**

For EHR‐derived data to yield reliable real‐world evidence, it needs to be of known and sufficiently high quality. Considering the impact of mortality data completeness on survival endpoints, we highlight the importance of data quality assessment and advocate benchmarking to the NDI.

Real‐world evidence (RWE) is championed as a complementary source to traditional clinical trial evidence (Sherman et al. 2016). RWE includes prospective and retrospective research (e.g., outcomes research, pragmatic clinical trials) based on data collected during routine clinical care. RWE results are more generalizable than traditional clinical trials because they represent the full distribution of patient and disease characteristics encountered. Data sources for RWE include electronic health records (EHRs), administrative claims data, and prospective registries. However, RWE depends on underlying data quality and the degree to which study design (including cohort selection) and analyses can reduce bias.

A key goal of medical evidence is to identify a survival benefit associated with a treatment regimen. Validity of the generated evidence hinges on quality of mortality variable(s) within the dataset. Mortality comprises key concepts of vital status (dead/alive), death date, and cause of death. In traditional clinical trials, considerable effort is dedicated to collecting mortality information to enable outcomes analyses. Within RWE, mortality information is frequently incomplete since data are not intentionally collected for research; this is especially true when source datasets are EHRs or administrative claims, but it may be less of a concern for prospective registries. Mortality data may be missing because of patients lost to follow‐up; imperfect mortality data capture systems; and clinical workflows not designed to collect mortality data.

To fill the resulting gap in real‐world mortality data, critical properties of a needed mortality data source include not only completeness and accuracy of the death variable(s), but also linkability to other data sources and timely availability of new data, here referred to as recency. Historically, RWE data sources have been supplemented with public mortality data, with the US Social Security Death Index (SSDI) being one of, if not the most, frequently used. However, following a 2011 statutory change, the public version of the SSDI no longer includes state‐sourced data, limiting completeness of these data upon which health care researchers have relied (US Department of Commerce 2011; Blackstone 2012; Sack 2012; da Graca, Filardo, and Nicewander 2013). While the NDI also comprises death records from state vital statistics offices, it is updated yearly. NDI's completeness makes it a good historical resource for benchmarking, but the annual release cadence limits its utility where data recency is important (Cowper et al. 2002; Blackstone 2012). Further, both the SSDI and the NDI have access restrictions that make them difficult to incorporate into all practical RWE use cases. Given these limitations of public U.S. death data, other mortality data sources with both high quality and recency are needed to enable accurate, timely outcomes research.

Mortality information for RWE data sources can be obtained and amalgamated in many ways. Commercial death datasets sourced from credit card, insurance, or obituary information can supplement public sources (Maynard 2013). Within EHR unstructured data, documentation of patient family communication may reveal that a patient died. EHR structured fields could be populated with mortality data based upon prompts or workflow changes. Regardless of the approach, mortality data quality in RWE sources must be characterized so that the strength of evidence can be assessed.

Here, we describe a composite dataset that leverages publically available SSDI and commercial death data to improve oncology‐specific EHR‐derived mortality data, benchmarked against the NDI at every step of dataset development. Novel aspects of this work include generation of an aggregate mortality data source and use of NDI as a national gold standard to evaluate data quality.

## Methods

### Overview of Design

The aim was to generate a composite mortality dataset by sequentially adding best available death data sources described below to resolve gaps in EHR‐derived data. Each dataset generated was benchmarked against the NDI using prespecified metrics defined below. In this work, we focused on vital status and death date, combined into a single date called “mortality variable”; if the death date was populated, then vital status was presumed “dead.” Cause of death was not considered. Death from any cause was included.

### Data Sources

Data sources used were as follows: (1) EHR structured data, contained within the Flatiron Health database; (2) abstracted data from EHR unstructured documents (e.g., end of treatment notes or condolence letters); (3) commercial death data purchased from a vendor (labeled CDD1); and (4) publicly available US mortality data, SSDI (Figure S1). NDI was used as the gold standard for benchmarking; NDI was not incorporated into the composite dataset because of usage restrictions and data time lag of over a year.

### Flatiron Health Database Description

During this analysis, the Flatiron Health database contained information from >260 cancer clinics including >1.6 million active U.S. cancer patients. These clinics use Flatiron Health's EHR (OncoEMR) or another EHR such as Epic; demographic, clinical, and outcomes data were extracted from EHR systems, including structured data and unstructured documents.

To create the database, patient‐level data were aggregated, normalized, and harmonized. Data were processed centrally and stored in a secure format. Structured data (e.g., treatments, laboratories, diagnoses) were semantically mapped to standard reference terminologies, where applicable. Death dates were from the EHR structured data field “patient date of death.” Unstructured data (e.g., medical care notes, death notices, condolence letters) were extracted from EHR‐based digital documents via “technology‐enabled” chart abstraction developed by Flatiron Health, which surfaces relevant documents to trained oncology abstractors. Every data point sourced from unstructured documents was manually reviewed by trained chart abstractors (oncology nurses/tumor registrars, with oncologist oversight).

Rigorous quality control (QC) included duplicate chart abstraction of a sample of critical abstracted variables. Additional QC was performed covering themes such as demographics and treatment length/dosage and included both medical considerations (e.g., expectations based on dataset‐relevant literature and clinical practice) and data considerations (e.g., expectations based on data source and physician documentation patterns). Identified issues were logged, prioritized, investigated, and resolved.

To link EHR structured data to external data sources (CDD1 and SSDI), a matching algorithm was developed based on available patient identifiers (e.g., first and last name, birthdate, state of residence). When a patient had a death date in two or all three sources, the two dates reported were identical for most cases (>92 percent). If all three or any two dates were the same, that date was used. Where the dates conflicted, the hierarchy for date selection was SSDI (deemed most reliable based on comparison to NDI), CDD1, and then EHR. Death dates known to be incorrect were removed (e.g., death dates prior to abstracted diagnosis dates).

### Cohort Selection

The “study cohort” consisted of a random sample of patients within the Flatiron Health analytic database with advanced non‐small‐cell lung cancer (advNSCLC). Advanced melanoma (advMelanoma), metastatic colorectal cancer (mCRC), and metastatic breast cancer (mBC) were included as a separate sensitivity analysis. These were selected to represent a range of cancer types, including cancers characterized by long versus short survival, cancers with substantial recent therapeutic advancements, and cancers treated in the community versus academic setting. The required sample size was calculated conservatively (assumed low event rates for each disease, 60 percent sensitivity, and 98 percent specificity). Therefore, if the sensitivity was estimated at 60 percent, this sample size allowed us to be 95 percent confident that the true sensitivity would be from 55 to 65 percent. Observed sensitivity was higher than our assumptions; therefore, our analysis yielded more precise estimates.

Cohort inclusion criteria were as follows: (1) cancer diagnosis, as documented by ICD‐9 or ICD‐10 code and confirmed in EHR unstructured data by trained chart abstractors to confirm the specific cancer type; (2) confirmation of advanced or metastatic disease by trained chart abstractors; (3) >1 oncologist visit on or after January 1, 2011; and (4) diagnosis of advanced/metastatic disease on or after January 1, 2011, and through December 31, 2015, for NSCLC, and from January 1, 2013, through December 31, 2015, for other tumor types. The December 31, 2015, cutoff was applied to align with NDI data availability.

### NDI Matching and Validation

An algorithm was developed to match study cohort patients to the NDI cohort (data available through December 31, 2015), as recommended in the NDI User's Guide (National Center for Health Statistics 2013). First, we noted study cohort patients with no death date in the NDI data and considered them alive (28 percent). Then, we identified true matches by finding those noted by NDI as exact matches (46 percent), and three other signs recommended by NDI that the match was likely a true match (6 percent). Next, we found patients who only had a death date that NDI noted is likely a false match and considered to be alive (17 percent). The remainder (3 percent) could not be classified and were conservatively considered to be alive.

Validation metrics included sensitivity, specificity, positive predictive value (PPV), negative predictive value (NPV), and date agreement (Table 1). As each death data source was added into the mortality variable, these metrics were assessed to understand the contribution of each to the composite.

**Table 1:** Validation Metrics for Mortality Data

		NDI data		
		Deceased	Alive	
Flatiron Health composite data	Deceased	True positives (A)	False positives (B)	PPV = A/(A + B)
	Alive	False negatives (C)	True negatives (D)	NPV = D/(C + D)
		Sensitivity = A/(A + C)	Specificity = D/(B + D)	

*Notes*.

For sensitivity and specificity analyses, an individual was placed into one of the four categories (A, B, C, or D), depending on how a patient's mortality status from the composite death date agreed with that from the NDI. True positives (A) were all individuals with a death date in both the composite dataset and the NDI. False positives (B) were all individuals with a death date in the composite dataset but not in the NDI. False negatives (C) were all individuals without a death date in the composite death dataset but with a death date in the NDI. True negatives (D) were all individuals who did not have a death date in the composite death dataset or in the NDI. Sensitivity indicated the percent of deaths in the NDI that were correctly recorded in the composite dataset, computed as the proportion of true positives among all the positives in the NDI gold standard [A/(A + C)]. Specificity indicated the percent of individuals without a death date in the NDI who were also not recorded as deceased in the composite dataset, computed as the proportion of true negatives among all the negatives in the NDI gold standard [D/(B + D)]. PPV indicated the percent of individuals with a death date in the composite dataset who were also considered dead in the NDI gold standard dataset [A/(A + B)]. NPV indicated the percent of individuals without a date of death in the composite dataset who were also not recorded as deceased in the NDI gold standard [D/(C + D)]. Date agreement indicated the percentage of the composite death dates that were exactly the same between NDI and the composite dataset; patients without a death in NDI but with a death in the composite dataset were counted as a disagreement in the date agreement calculation. Date agreement was also calculated allowing for a ±15‐day window and a ±30‐day window.

### Analyses

Subgroup analyses were performed to identify any differences in sensitivity of the variable based on key characteristics. This included subgroup analyses by practice (defined as a financial entity and may include multiple sites of care) for practices with ≥100 patients, and over time for the advNSCLC cohort since we analyzed deaths in the 2011–2015 time period as well as restricted to the same time period as for the rest of the tumor types (2013–2015).

Kaplan–Meier survival analyses were generated using all sets of mortality data to estimate comparative times to event using different composite death datasets versus NDI.

### Ethical Considerations

Institutional Review Board (IRB) approval of the study protocol was obtained. Informed consent was waived by the IRB as this was a noninterventional study using routinely collected data. This study was also reviewed and approved by the National Center for Health Statistics, a division of U.S. Centers for Disease Control, which oversees the NDI. Flatiron Health standard methodology for data security and patient privacy were implemented for this work.

## Results

### Mortality Variable Quality in EHR Structured Fields

We characterized mortality variable quality in the EHR‐derived structured data (here referred to as EHR) for the advNSCLC cohort (Table 2) by benchmarking against NDI death data. Sensitivity was low (66 percent), but specificity was high (97 percent). Exact death date agreement was 89 percent. Since EHRs lack standardized processes to collect death dates, we also determined ±15 and ±30 day agreement, which was much higher (97 and 97 percent, respectively). Table S1 shows the contingency table with the overlap between NDI data and the EHR only mortality variable.

**Table 2:** Validation Metrics during Each Step in the Development of the Mortality Variable for the Advanced NSCLC Cohort

	Sensitivity	Specificity	PPV	NPV	Date Agreement (Exact Date)	Date Agreement (±15 days)	Date Agreement (±30 days)
Structured EHR only (EHR)	65.97% (64.84%, 67.09%)	97.06% (96.49%, 97.63%)	97.82% (97.40%, 98.24%)	58.78% (57.50%, 60.07%)	88.70% (87.72%, 89.67%)	96.53% (95.99%, 97.07%)	96.99% (96.49%, 97.49%)
SSDI only	34.73% (33.59%, 35.86%)	99.06% (98.73%, 99.38%)	98.66% (98.20%, 99.12%)	43.15% (42.05%, 44.25%)	97.28% (96.62%, 97.94%)	98.45% (97.95%, 98.95%)	98.49% (98.00%, 98.99%)
EHR‐CDD1	84.06% (83.19%, 84.93%)	96.26% (95.63%, 96.90%)	97.83% (97.45%, 98.20%)	75.13% (73.85%, 76.42%)	92.33% (91.62%, 93.04%)	96.87% (96.41%, 97.32%)	97.41% (97.00%, 97.83%)
EHR‐CDD1 + SSDI	88.83% (88.08%, 89.58%)	96.06% (95.40%, 96.71%)	97.83% (97.46%, 98.19%)	81.14% (79.93%, 82.35%)	93.83% (93.21%, 94.45%)	97.07% (96.64%, 97.49%)	97.52% (97.13%, 97.91%)
EHR‐CDD1‐SSDI + ABS (Final v2.0)	90.60% (89.90%, 91.29%)	96.00% (95.34%, 96.66%)	97.84% (97.48%, 98.20%)	83.62% (82.46%, 84.78%)	93.50% (92.87%, 94.13%)	97.00% (96.57%, 97.42%)	97.49% (97.10%, 97.88%)

*Notes*.

For each step in the variable development process, 95% CIs are shown. This cohort included patients diagnosed with advanced NSCLC on or after January 1, 2011, and through December 31, 2015 (*N* = 10,195).

### Supplementing EHR Data to Create a Composite Dataset

To create a consensus dataset with greater sensitivity and date agreement than the EHR data alone, we selected a commercial dataset on the basis of data coverage and recency (CDD1). Following linking the EHR‐based dataset and CDD1, sensitivity of the combined EHR‐CDD1 dataset in advNSCLC increased (66 to 84 percent), along with exact date agreement (89 to 92 percent), without a major effect on specificity (97 to 96 percent; Table 2). Next, despite only 35 percent sensitivity of SSDI data alone for this cohort, adding SSDI to EHR‐CDD1 further increased sensitivity from 84 to 89 percent.

Finally, we considered the resource‐intensive solution of abstracting patient charts where death date was not available elsewhere and who did not have recent activity (e.g., in the past 60 days). Adding dates of death abstracted from EHR unstructured fields (ABS) increased sensitivity by an additional 2 percent, to 91 percent (EHR‐CDD1‐SSDI‐ABS; Table 2). Table S2 shows the contingency table for the composite mortality variable. This final dataset comprises the advNSCLC composite mortality variable (version 2.0; available in all Flatiron Health datasets after June 2017).

### Effect of Mortality Data Completeness on Outcomes

**Figure 1:**
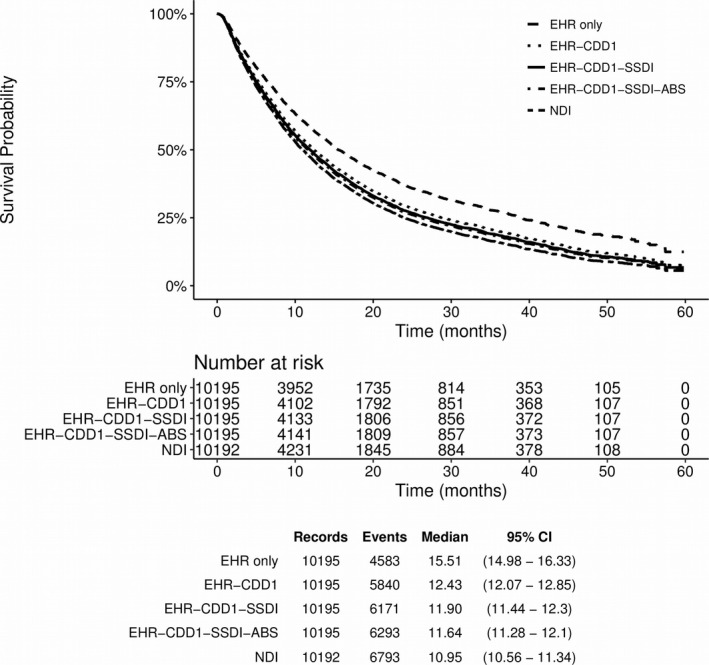
Overall Survival for Advanced NSCLC Determined Using Indicated Mortality Data 

*Notes*. NDI data were used as the benchmark in this study and were assumed to have 100 percent completeness. Patients were excluded from this analysis if their death date fell before the advanced diagnosis date.

Mortality data quality impacts survival estimates and other endpoints relying on this variable (e.g., progression‐free survival). Using the datasets of varying completeness created en route to the advNSCLC composite mortality dataset, we determined how overall survival is impacted by mortality data quality (Figures 1 and S2). Overall survival was also calculated for NDI data only, which was assumed to have 100 percent completeness.

### Sensitivity Variability in Individual Clinical Practice Data

**Figure 2:**
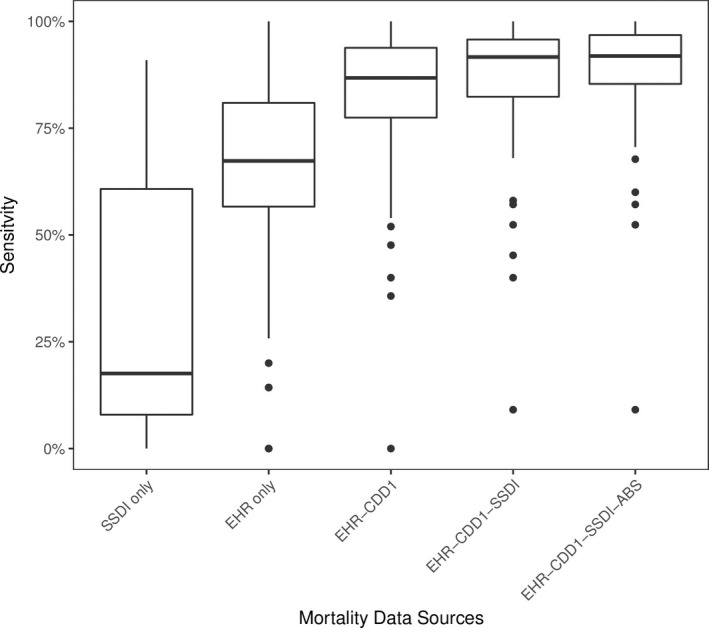
Sensitivity of advNSCLC Data by Practice 

*Notes*. Data were restricted to practices with ≥100 patients. Boxplots show the median sensitivity, with lower and upper hinges of the boxes corresponding to the 25 and 75 percent interquartile range (IQR); lower and upper whiskers indicate sensitivity within 1.5 IQR of the lower and upper quantiles, respectively; and points outside of the whiskers show the rest of the data.

Next, we analyzed variability in all advNSCLC mortality datasets for unique oncology practices in the EHR dataset, because documentation and data entry patterns may differ by practice, resulting in differences in EHR structured and unstructured data quality. Variability of sensitivity values narrowed as each new data source was added (Figure 2). This analysis was limited to practices with at least 100 patients, to reduce the impact of small practices with only a few deaths, which due to small sample size may have very high or low sensitivity. Each incrementally added dataset filled gaps in the data; for example, the 25th percentile for data completeness in the EHR only dataset was 57 percent, increasing to 85 percent once all data sources of were amalgamated. This highlights the importance of amalgamating sources with different strengths, weaknesses, and areas of missingness.

There were some patterns we observed in the EHR data that may indicate sporadic errors in the recording of data in clinical practice. For example, in some cases, we observed a date of death in the EHR that is exactly 1 year from the NDI date of death. This could potentially indicate an error such as the provider mistyping the year of death into the EHR. However, the high date agreement observed (97 percent within 15 days of the NDI date of death) demonstrates that these potential errors are occurring in a very small percentage of cases. Future solutions include workflow prompts in the EHR that encourage accurate data entry, such as date of death triggering production of a condolence card that clinic staff sign and send to the family.

### Composite Mortality Datasets in Different Tumor Types

Using the methodology described above, we assembled datasets for advMelanoma, mCRC, and mBC. Sensitivity was ≥85 percent for all datasets, with small differences in data quality by tumor type (Table 3).

**Table 3:** Validation Metrics for Different Tumor Types, with Data Shown for the Final Mortality Variable That Comprises Structured EHR Data, CDD1, SSDI, and Abstraction of Unstructured EHR Data

	Sensitivity	Specificity	PPV	NPV	Date Agreement (Exact)	Date Agreement (±15 days)	Date Agreement (±30 days)
advNSCLC (*N* = 6,840)	89.70% (88.76%, 90.64%)	97.30% (96.70%, 97.89%)	97.89% (97.43%, 98.36%)	87.09% (85.92%, 88.25%)	93.38% (92.55%, 94.22%)	96.96% (96.40%, 97.53%)	97.54% (97.03%, 98.05%)
advMelanoma (*N* = 1,622)	88.39% (85.97%, 90.81%)	98.84% (98.16%, 99.52%)	98.18% (97.12%, 99.25%)	92.33% (90.69%, 93.97%)	95.37% (93.66%, 97.09%)	97.36% (96.06%, 98.65%)	97.85% (96.68%, 99.02%)
mCRC (*N* = 7,325)	85.26% (83.97%, 86.54%)	98.23% (97.84%, 98.62%)	96.97% (96.30%, 97.63%)	90.93% (90.12%, 91.75%)	91.95% (90.85%, 93.05%)	95.84% (95.05%, 96.63%)	96.69% (95.99%, 97.40%)
mBC (*N* = 3,792)	86.95% (85.11%, 88.80%)	98.49% (98.01%, 98.96%)	96.70% (95.67%, 97.73%)	93.68% (92.75%, 94.60%)	91.40% (89.70%, 93.09%)	95.83% (94.65%, 97.01%)	96.26% (95.15%, 97.38%)

*Notes*.

The cohorts here included patients with the respective diagnoses on or after January 1, 2013, and through December 31, 2015, as data for advMelanoma, mCRC, and mBC were available from this date. The advNSCLC cohort was restricted to the same date range here to enable comparisons of data across the cohorts. 95% CIs are shown.

## Discussion

The EHR is championed as an important real‐world data source (Khozin, Blumenthal, and Pazdur 2017). However, there are clear gaps in critical data points like vital status and death date. In prospective clinical studies, these variables are intentionally collected, with presumed near‐complete data. Reliability of EHR data for retrospective and prospective RWE requires resolution of data gaps to the greatest extent possible and characterization of dataset quality. To resolve data gaps, nationally available data sources like the SSDI and NDI have been used, but challenges with deteriorating data completeness (SSDI), recency (NDI), and accessibility (SSDI, NDI) have limited their utility for the full breadth of contemporary RWE studies.

Here, we describe both an approach to compiling progressively more complete EHR‐based mortality data, as well as benchmarking data quality against a prespecified gold standard. By amalgamating EHR and other data sources, sensitivity of the mortality information for patients with advNSCLC improved from 66 to 91 percent, and survival estimates overlapped with estimates based upon NDI data.

If EHR data represent a complete clinical case history for a patient, why would mortality data be incomplete? First, EHRs are tools used by hospitals, health systems, or clinical practices and thus belong to and serve that system. Patients who leave the system may enter hospice, receive treatment elsewhere, or move away are lost to follow‐up. Second, clinical trials spend considerable effort following patients, but this is not a part of routine clinical care workflows, making real‐world mortality data inherently less complete. Following a patient's death, there is not usually a reimbursement‐related or clinical need for documentation; the main mandate for mortality data in clinical practice might simply be to send a condolence letter—or nothing. Third, there is huge variation in documentation patterns between clinical care providers, potentially resulting in incomplete or imprecise patient data.

The goal of this work was to create a real‐world composite mortality dataset of sufficient quality for retrospective and prospective studies that leverage RWE. A retrospective RWE study might compare overall survival of two options for first‐line management of advNSCLC. In a prospective clinical trial, real‐world EHR data might fill in the majority of the study dataset, or EHR data might be used to monitor long‐term survival follow‐up in a clinical trial that has completed assessment of its progression‐free survival primary endpoint. For these and many other use cases, sufficiently high data quality is needed for RWE credible enough to inform and potentially change clinical care.

A remaining fundamental question is “what is good enough?” Discussions with experts in the field suggest a sensitivity threshold of 90 percent, in line with what we observed for the composite advNSCLC dataset. Comparison of survival curves with the NDI reinforces this assertion. Subsequent work will assess mortality data quality in additional tumor types as well as determine the impact and limitations of the composite datasets for additional retrospective and prospective use cases. We have begun an assessment of informative censoring considering that there was some missing death data in the Flatiron Health mortality dataset; although we observed an effect, it was of small magnitude and did not substantively impact conclusions (manuscript in progress). Last, while some data sources have death information within 7 days of the death (e.g., CDD1), we plan to investigate when data can be considered adequately mature for various analyses, as deaths may not be recorded immediately.

Other means of improving data quality continue to be sought. The NDI may be considered when the research question can accommodate a time lag. The NDI‐benchmarking framework outlined here can be used to understand the impact of integrating new mortality datasets. EHRs can improve clinical workflows (e.g., encouraging physicians to send condolence cards) and in turn improve mortality data capture. And, as has been recommended in many fora, public policy should support the development of a nationally available, timely death dataset.

The framework described here was specifically developed to address challenges of accessing and linking U.S. mortality data for this oncology‐specific dataset. In international settings, such linking would be easier in cases where comprehensive death data are available within single‐payer health care systems, but a similar composite, stepwise dataset development process can be used when complete and timely national data are not available. While we do not have data for diseases other than the cancer types studied here, for diseases other than oncology, completeness and accuracy of EHR‐captured mortality data would be dependent on whether death data collection is integrated into the care workflow. Combining any EHR‐derived data with commercial and national data sources that capture all‐cause mortality even after the patient may be lost to follow‐up at the particular site of care is important to increase the completeness of the combined dataset. This will be particularly important for chronic diseases where patients are more likely to be lost to follow‐up. Finally, the key step for any dataset is the designation of a standard benchmark to enable measurement of mortality data completeness such as the NDI used here.

This work has some important limitations. First, we used the NDI as the gold standard; thus, all validation metrics depend on the quality and recency of NDI data. However, the NDI data might not be 100 percent complete in all cases, as previously reported (Calle and Terrell 1993). Via chart review of a sample of 20 false positives where the EHR had a death date that NDI did not have within the same time period, one patient was confirmed as deceased, one patient was confirmed as not deceased (the EHR date of death was entered incorrectly), 8 others were transferred to hospice or stopped care, indicating that the EHR‐derived mortality data were likely correct, and 10 were unknown. In another chart review of 20 false positives where CDD1 or the SSDI had a death date that NDI did not have within the same time period, 11 were confirmed dead, 6 had a referral or were admitted to hospice within a month of the external date of death, and 3 were unknown. These may be missing in the NDI due to imperfect sensitivity, or because of gaps in the matching algorithm between our data and the NDI. While we used the recommendations of the NDI user's guide to match patients, 3 percent of patients were not able to be classified, and these patients may also be included in these false positives. Second, the lack of NDI recency does not allow us to validate data after 2015; while we believe that the CDD1 data have improved over time, we cannot test this hypothesis until new updates of the NDI are available. Third, as reported in literature (Blackstone 2012; Sack 2012; da Graca, Filardo, and Nicewander 2013), SSDI quality declines may lead to attenuated contribution to the composite mortality dataset in the future. Fourth, the mortality variable does not include cause of death. Cause of death is less important for many cancer‐related RWE studies but may be important for certain research questions and in other therapeutic areas. Also, combining vital status and death date into a single variable limits our ability to indicate that a patient is deceased but the date is unknown. Finally, the benchmarking described here did not include academic centers due to data availability limitations; those data may differ in sensitivity. We will continue to monitor data quality over time and evaluate additional sources of death data.

By combining multiple datasets, we increased the sensitivity of the mortality dataset to 85 to 91 percent while maintaining high specificity (>97 percent) across the four cancer types benchmarked against NDI. The high quality and recency of this variable make it suitable for evaluating outcomes in oncology using retrospective and prospective study designs that leverage RWE. While this work focused on an example in oncology, the same framework and validation approach could be used across disease areas.

Although there are clear advantages to amalgamating multiple sources for death data, there are also challenges that arise when linking patients between sources. We linked EHR data to two external data sources (CDD1 and SSDI) using a matching algorithm. While we have confidence in our approach due to the resulting high sensitivity, specificity, and date agreement with the NDI, patient misidentification is still possible, as may more likely be the case when patients move, change names, or have common names. This may lead to incorrectly specifying someone as dead, or missing a true death which highlights the importance evaluating the quality of the approach against a gold standard, as was done here.

More broadly, as RWE gains use in supporting evidence‐based care and regulatory decisions (Sherman et al. 2016), it is particularly important to set quality standards for variables such as mortality and define what constitutes sufficiently high quality for various use cases. In parallel to this work, it is also important for public policy to support the development of a complete and timely national mortality resource available to facilitate generation of reliable and actionable RWE.

## Supporting information

Appendix SA1: Author Matrix.

Figure S1: Death Data Sources Used in Developing the Composite Mortality Datasets Investigated.
Figure S2: Overall Survival for Advanced NSCLC Determined Using Indicated mortality Data, as in Main Text Figure 1, with Censoring Marks Added.
Table S1: Validation Metrics for the Structured EHR Only (EHR) Mortality Variable in the advNSCLC Patient Cohort.
Table S2: Validation Metrics for the Complete Composite Mortality Variable (EHR‐CDD1‐SSDI‐ABS) in the advNSCLC Patient Cohort.
