# Supplementary material for: Development and Validation of a High‐Quality Composite Real‐World Mortality Endpoint
Source: Health Serv Res. 2018 May 14;53(6):4460–76. doi: 10.1111/1475-6773.12872 (PMC6232402; doi:10.1111/1475-6773.12872)
Supplement: Supplementary file 2 — Figure S1: Death Data Sources Used in Developing the Composite Mortality Datasets Investigated. Figure S2: Overall Survival for Advanced NSCLC Determined Using Indicated mortality Data, as in Main Text Figure 1, with Censoring Marks Added. Table S1: Validation Metrics for the Structured EHR Only (EHR) Mortality Variable in the advNSCLC Patient Cohort. Table S2: Validation Metrics for the Complete Composite Mortality Variable (EHR‐CDD1‐SSDI‐ABS) in the advNSCLC Patient Cohort. [file HESR-53-4460-s002.pdf]

SUPPLEMENTAL MATERIAL

Development and validation of a high-quality composite real-world mortality endpoint

Melissa D. Curtis; Sandra D. Griffith; Melisa Tucker; Michael D. Taylor; William B. Capra; Gillis Carrigan; Ben Holzman; Aracelis Z. Torres; Paul You; Brandon Arnieri; Amy P. Abernethy

Supplemental Table 1. Validation metrics for the structured EHR only (EHR) mortality variable in the advNSCLC patient cohort.

		NDI data		
		Deceased	Alive	
**Structured EHR only (EHR)**	Deceased	4483 (True positives)	100 (False positives)	97.82% (PPV)
	Alive	2313 (False negatives)	3299 (True negatives)	58.78% (NPV)
		65.97% (Sensitivity)	97.06% (Specificity)	

Supplemental Table 2. Validation metrics for the complete composite mortality variable (EHR-CDD1-SSDI-ABS) in the advNSCLC patient cohort.

		NDI data		
		Deceased	Alive	
**EHR-CDD1-SSDI-ABS (final v2.0)**	Deceased	6157 (True positives)	136 (False positives)	97.84% (PPV)
	Alive	639 (False negatives)	3263 (True negatives)	83.62% (NPV)
		90.60% (Sensitivity)	96.00% (Specificity)	

Supplemental Figure 1. Death data sources used in developing the composite mortality datasets investigated.

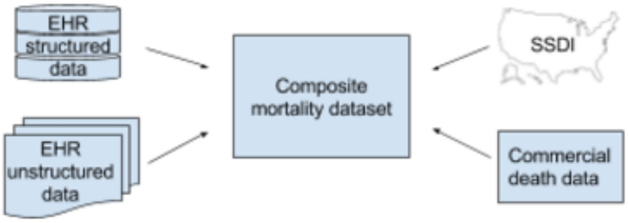

Supplemental Figure 2. Overall survival for advanced NSCLC determined using indicated mortality data, as in main text Figure 1, with censoring marks added.

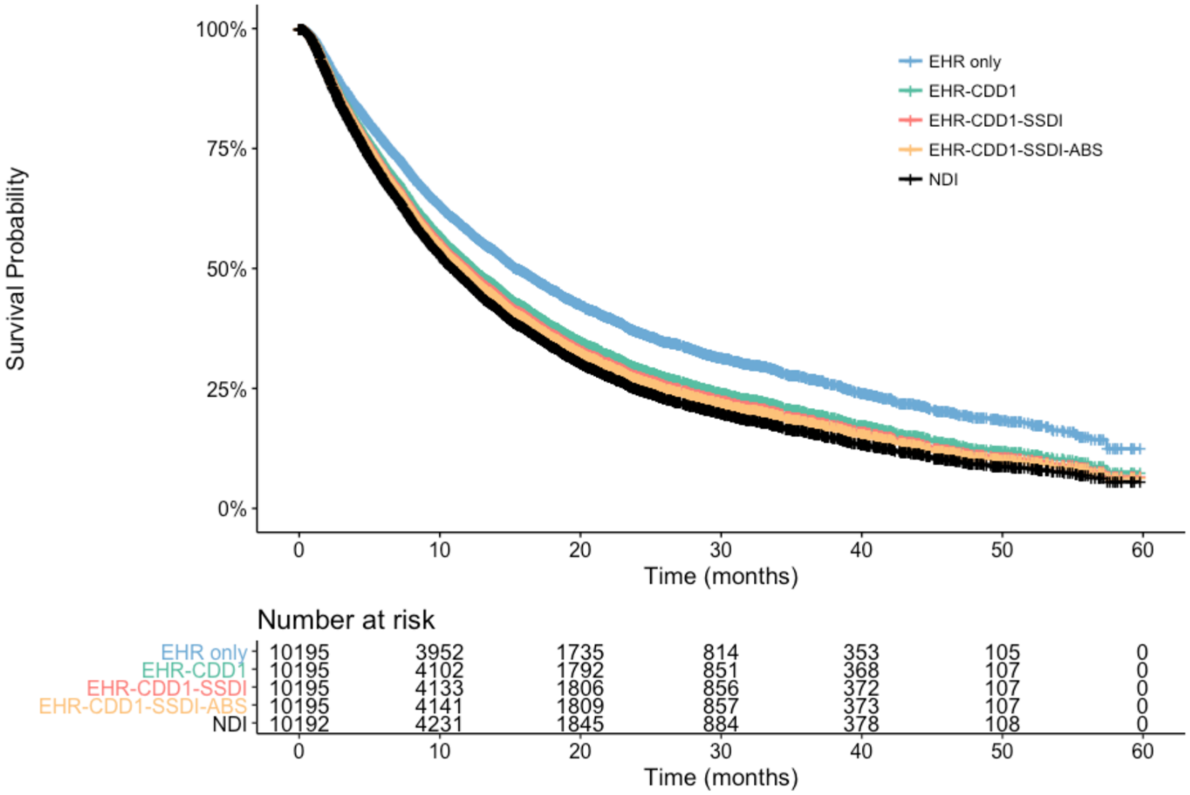

	Records	Events	Median	95% CI
EHR only	10195	4583	15.51	(14.98 - 16.33)
EHR-CDD1	10195	5840	12.43	(12.07 - 12.85)
EHR-CDD1-SSDI	10195	6171	11.90	(11.44 - 12.3)
EHR-CDD1-SSDI-ABS	10195	6293	11.64	(11.28 - 12.1)
NDI	10192	6793	10.95	(10.56 - 11.34)
